# Integrated microRNA-mRNA analyses reveal OPLL specific microRNA regulatory network using high-throughput sequencing

**DOI:** 10.1038/srep21580

**Published:** 2016-02-12

**Authors:** Chen Xu, Yu Chen, Hao Zhang, Yuanyuan Chen, Xiaolong Shen, Changgui Shi, Yang Liu, Wen Yuan

**Affiliations:** 1 Department of Orthopedics, Changzheng Hospital Affiliated to Second Military Medical University, 415th Feng Yang Road, Shanghai 200003, PR China

## Abstract

Ossification of the posterior longitudinal ligament (OPLL) is a genetic disorder which involves pathological heterotopic ossification of the spinal ligaments. Although studies have identified several genes that correlated with OPLL, the underlying regulation network is far from clear. Through small RNA sequencing, we compared the microRNA expressions of primary posterior longitudinal ligament cells form OPLL patients with normal patients (PLL) and identified 218 dysregulated miRNAs (FDR < 0.01). Furthermore, assessing the miRNA profiling data of multiple cell types, we found these dysregulated miRNAs were mostly OPLL specific. In order to decipher the regulation network of these OPLL specific miRNAs, we integrated mRNA expression profiling data with miRNA sequencing data. Through computational approaches, we showed the pivotal roles of these OPLL specific miRNAs in heterotopic ossification of longitudinal ligament by discovering highly correlated miRNA/mRNA pairs that associated with skeletal system development, collagen fibril organization, and extracellular matrix organization. The results of which provide strong evidence that the miRNA regulatory networks we established may indeed play vital roles in OPLL onset and progression. To date, this is the first systematic analysis of the micronome in OPLL, and thus may provide valuable resources in finding novel treatment and diagnostic targets of OPLL.

Ossification of the posterior longitudinal ligament (OPLL) is a genetic disorder which involves pathological heterotopic ossification of the posterior longitudinal ligament. The disease is commonly found in Asian people with the incidence of 2.4%, and twice as common in men as in women^1^,^2^. The ossified ligaments formed osteophytes that gradually increases in size, which in turns causes compression of spinal cord and may lead to neurologic symptoms or severe neurologic deficit. Recent studies have shown that single nucleotide polymorphisms (SNPs) and gene expression variations in various genes are associated with OPLL development^3^,^4^,^5^,^6^,^7^, which implies genetic factors may play key roles in this process. However, the exact pathogenesis of OPLL still remains unclear, not to mention the underlying mechanism and its regulatory network.

Among the critical genes that were reported to be associated with the development of OPLL, many of them were also in association with other biological ossification processes, one such example is Runx2. Although Runx2 was shown to be closely related to OPLL development, but it also participates in many other ossification related events such as chondrocyte maturation, endochondral ossification and fetal bone development^8^,^9^,^10^. Concerning this fact, much has to be done in defining OPLL specific genes and their regulatory network.

MicroRNAs (miRNAs) are small (~21 nucleotides long) non-coding RNAs that participate in numerous biological or pathological processes of various regions. The function of which are to degrade target mRNAs or repress their translation by binding with the complementary regions in the mRNA molecules^11^. In the field of ossification, many studies have proved the important roles of microRNAs in regulating this biological process in various tissues^12^,^13^,^14^. However, less is known about the significance of microRNAs in the pathological process of posterior longitudinal ligament ossification.

Since many ossification related genes were reported to be associated with the development of OPLL, their regulatory upstream factors seemed to be of importance in defining the OPLL specific regulatory mechanism. MicroRNA is one such factor that worth exploring. Although the total number of defined microRNA molecules are no more than a few thousands, but they can regulate numerous gene expressions and even change the state of cells^15^. Moreover, under the consensus that one microRNA can target various mRNA that harbor target sites, and one mRNA can receive regulations from many microRNAs, global microRNA regulatory network analysis seem to be necessary and helpful in investigating the microRNAs’ functions in OPLL. In order to decipher the upstream regulatory networks in OPLL, here we take the advantage of high throughput sequencing and bioinformatics technology, and presented an integrative analysis of the whole transcriptome and its regulatory miRNA networks in OPLL compared with normal posterior longitudinal ligament (PLL) patient primary cell samples. We identified differentially expressed miRNAs and compared this expression pattern with multiple high throughput data from various cell types. We found most differentially expressed miRNAs in OPLL are cell type specific. Furthermore, the analysis of OPLL specific miRNA/mRNA interacting network showed key miRNA/mRNA interacting pairs that correlated with ossification and skeletal development, which is vital to OPLL development. Taken together, our findings suggest that altered OPLL specific miRNAs expressions may be responsible to OPLL development for its predicted regulatory genes closely related to ossification. Moreover, our data may also provide novel diagnostic and therapeutic interventional targets in OPLL with further validation.

## Results

### MicroRNAs were differentially expressed in OPLL derived primary ligament cells compared to normal primary posterior longitudinal ligament cells

In order to study the global microRNA (miRNA) expression change in OPLL compared with PLL, we first obtained tissue samples from cervical spine corpectomy patients either with ossified posterior longitudinal ligament (OPLL, n = 3) or normal posterior longitudinal ligament (PLL, n = 3). Obtained tissues were subjected to primary ligament cell culture immediately, and only ligament cells from passage 1 were used in the high throughput profiling analysis. After the sequencing, approximately 1,000,000 clean reads per sample were generated. All reads were mapped with annotated miRNAs in miRBase database (version 20), and approximately 35% of the clean reads were mapped to mature miRNA in the database, while approximately another 35% of the clean reads were mapped to ribosome RNA (rRNA) or Small nucleolar RNAs (snoRNA) when mapped to other small RNA datasets.

**Figure 1:**
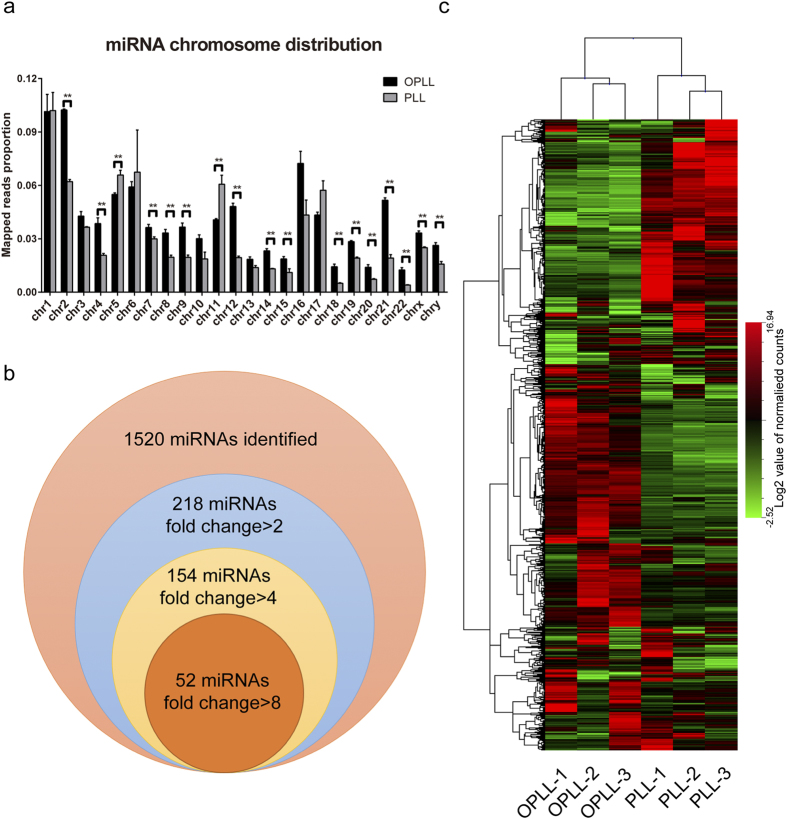
MicroRNAs were differentially expressed in OPLL derived primary ligament cells. The sequenced miRNA reads were mapped to the human genome (hg19), and the average percentage of reads distributed in each chromosome were analyzed and shown (**a**). A Venn diagram (**b**) showing the number of total mapped miRNAs in both groups and the number of differentially expressed miRNAs between two groups. Hierarchical clustering (**c**) of both samples and the miRNAs were performed to visualize the miRNA expression patterns in two groups. Each column represents each sample indicated in the bottom, each row represents an identified miRNA. The expression levels are depicted according to the color scale (middle right). Red or green indicate expression levels above or below the median, respectively. The magnitude of deviation from the median is represented by color saturation.

Comparing the results of OPLL and PLL group, significant difference in mapped reads’ chromosome distribution were found initially (Fig. 1a), which gave direct evidence of altered global miRNA expression between the two groups. After analyzing and normalizing all the mapped reads in both groups, we identified the existence of 1520 miRNAs altogether. After applying a stringent filtering critiria that compared OPLL with PLL (False discover rate, FDR < 0.01, fold change >2 or <0.5), we identified 144 upregulated and 74 downregulated miRNAs (Fig. 1b, Table 1). Hierarchical cluster analysis was performed based on these data using Euclidean distance similarity metric to test the similarity of these samples in a global level (Fig. 1c). A heatmap of all 1520 detected miRNAs in two groups were generated, and showed distinct miRNA expression pattern between two groups. And the clustering of samples showed that samples in the same group were much alike as the two groups were divided initially in the cluster tree. Taken together, by comparing the mirNome expression data of OPLL and PLL primary ligament cells, we showed significant difference exists in the miRNA expression between the two groups.

### Defining OPLL specific microRNA signatures through multiple microRNA profiling comparison

Although altered miRNAs were found in OPLL using high throughput approaches, but the specificity of these miRNAs have not been tested, especially in the context that OPLL may share common traits with other ossification processes. To clarify and define OPLL specific miRNAs, we selected multiple miRNA profiling data from the Gene Expression Omnibus repository (GEO, http://www.ncbi.nlm.nih.gov/gds). We searched for miRNA profiling data from cell types that resemble posterior longitudinal ligament cells, and chose GSE19232^16^ (Contains miRNA data of dermal fibroblasts, mesenchymal stem cells and osteo-differentiated mesenchymal stem cells), GSE34144^17^ (Contains miRNA data of osteoblasts), GSE47025^18^ (Contains miRNA data of dental pulp cells and periodontal ligament cells) for further analysis.

**Figure 2:**
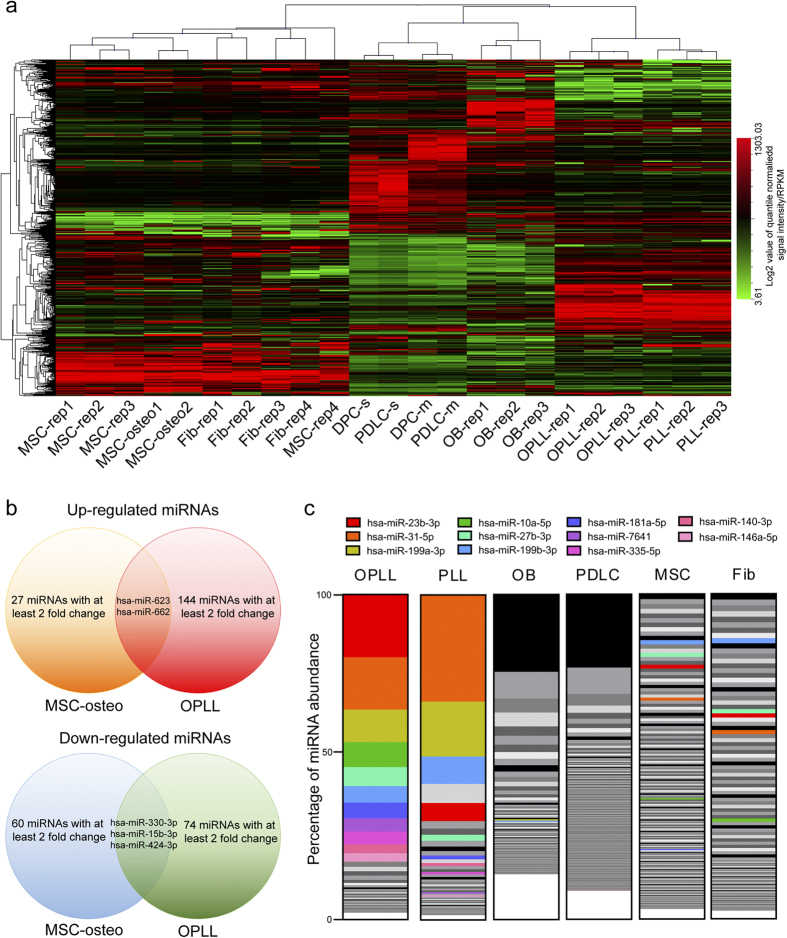
The differentially expressed microRNAs are specific to OPLL. (**a**) Unsupervised hierarchical clustering of miRNA profiling data of multiple cell to analysis the miRNA expression pattern in each cell type. Each column represents each sample indicated and each row represents a miRNA. The expression levels are depicted according to the color scale (middle right). The differentially expressed miRNAs in OPLL were further analyzed for specificity compared with the differentially expressed miRNAs in osteoblast induced MSCs, which share common traits with the ossification of PLL. The Venn plots (**b**) showed the overlapped miRNAs between two differentially expressed miRNAs in two groups. The abundance differences of OPLL expressed miRNAs in multiple cell types were shown in a stacked bar graph (**c**). Each vertical bar represents one cell type, and each block in the bar represents the expression abundance of one miRNA (The abundance is shown in percentage of the overall miRNA expression abundance, TPM or intensity value form the profiling data were used as expression abundance). MiRNA expression abundance in each sample was first ranked and showed from the highest (top of the bar) to the lowest (bottom of the bar). The color labelled blocks represent the top 11 OPLL abundant miRNAs. A more detailed ranking profile of OPLL differentially expressed miRNAs can be found in Supplementary Data 1.

To further analyze the concordance of different miRNA profiling datasets, we first normalized these data using quantile normalization method. After normalization, Hierarchical cluster analysis was performed. The results showed that although samples tend to be clustered according to the utilized platform, but the up or down regulated miRNAs were consistently different between cell types (Fig. 2a). The results indicated that OPLL altered miRNAs may be specific to OPLL, as no significant similarity were found between other cell types. However, detailed identification are performed to further specify these microRNAs.

Mesenchymal stem cells (MSCs) are pluripotent cells that can differentiate into mature osteoblasts, and the differentiation process is also well characterized. To test whether OPLL related miRNA changes also take place in the osteo-differentiation of MSCs, we compared the upregulated and downregulated miRNAs in the two datasets. In the comparison, of the 124 miRNAs that were upregulated in the OPLL, only 2 were overlapped with MSC/osteo transition (Fig. 2b). In the downregulated miRNAs, 3 out of 75 miRNAs were found overlapping (Fig. 2b). To gain more evidence on the specificity of these dysregulated miRNAs, we compared their abundance of each cell type. We first ranked all miRNAs by their profiling expression in every cell type, and compared their rank with each cell type. We found that the most abundant miRNAs (Colour labeled) in OPLL make up approximately 80% of the total miRNA abundance (Normalized counts), and most of them still compromise more than 70% total miRNA abundance in the PLL (Fig. 2c). However, this expression pattern is not found in either osteoblasts or periodontal ligament cells. In the case of MSC and fibroblasts, although some miRNAs have similar rank places in OPLL, but the overall state is still more dissimilar (Fig. 2c). The initial result indicated that from the aspect of global miRNA expression, every cell type we tested is unique from each other. Comparing the rank place changes of formerly discovered OPLL dysregulated miRNAs in these cell types, we found over 50% of them do not have similar rank places in cell types other than OPLL or PLL (supplementary data 1). Taken together, although some OPLL altered microRNAs share common expression traits with osteo-differentiation of MSCs or fibroblasts, the majority of identified OPLL altered miRNAs are OPLL specific (Table 1).

### Global transcriptome analysis revealed OPLL altered gene expressions are related to ossification and skeletal development

**Figure 3:**
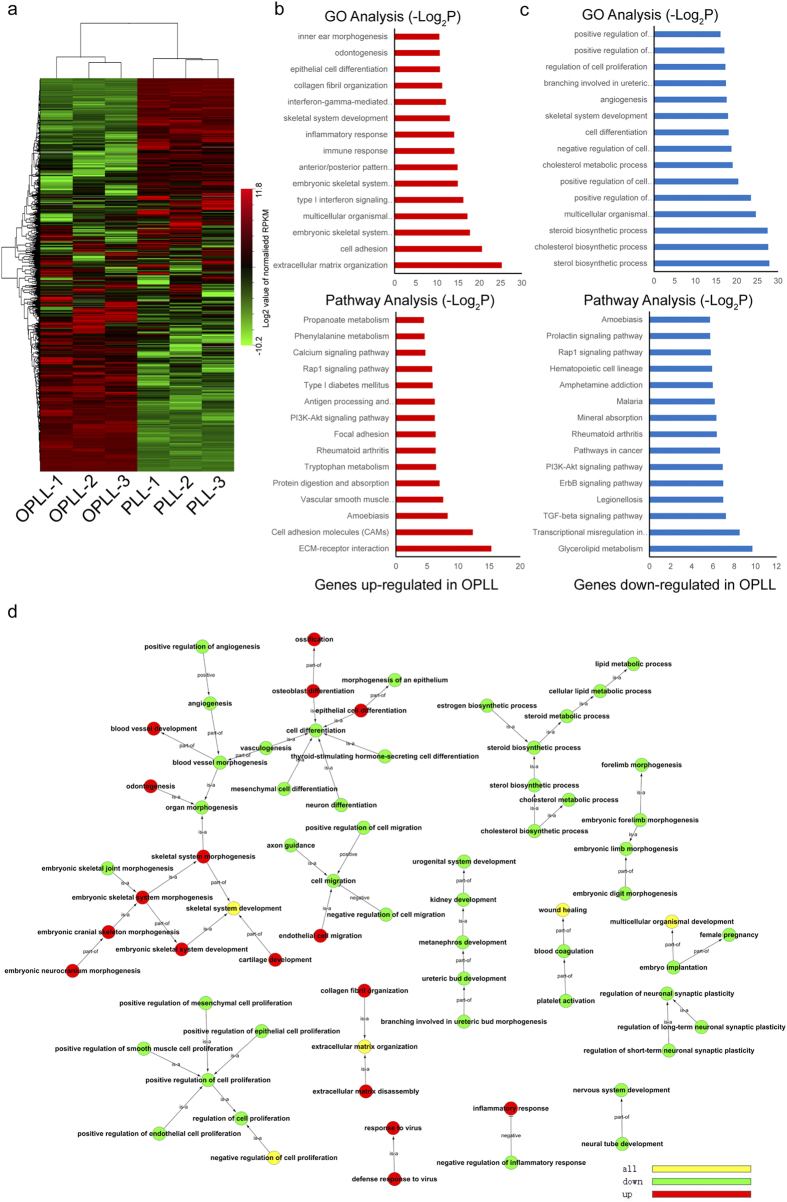
Genes were differentially expressed and related to ossification in OPLL. The global transcriptome of OPLL (n = 3) and PLL (n = 3) were analyzed and the unsupervised hierarchical clustering of the mRNA that detected in each sample were shown (**a**). Each column represents each sample indicated and each row represents a miRNA. The expression levels are depicted according to the color scale (middle right). To fully characterize these differentially expressed genes, Gene Ontolgy (GO) (**b**) and KEGG pathway analysis (**c**) were employed. GO or pathway terms in red bars represent analysis of upregulated genes in OPLL, while blue bars represent analysis of genes down regulated in OPLL. The enriched GO terms were sequentially constructed into a GO tree diagram (**d**) identifying the relationship of upregulated genes, downregulated genes or ambiguously enriched GO terms.

To fully decipher the OPLL miRNA regulatory network, we first analyzed the global transcriptome in both OPLL and PLL. Taking advantage of Hierarchical cluster, we found the global gene expression is greatly altered (Fig. 3a), with 1667 genes significantly upregulated (Fold change >2, FDR < 0.05) and 1264 genes significantly downregulated (Fold change <0.5, FDR < 0.05). In order to categorize the altered genes, we applied Gene Ontology (GO) analysis. Altogether 65 significant (P < 0.01) GO terms were associated with upregulated genes, among which extracellular matrix organization, embryonic skeletal system morphogenesis, embryonic skeletal system development, skeletal system development, collagen fibril organization, osteoblast differentiation, ossification are more significant and highly enriched (Fig. 3b, Supplementary data 2). However, these terms were not found in the GO analysis in genes downregulated in OPLL (Fig. 3c). In the Kegg pathway analysis of altered genes, we also found that ECM-receptor interaction and Calcium signaling pathway are highly enriched in upregulated genes, while Mineral absorption related pathway and Glycerolipid metabolism are enriched in the downregulated genes. Taken these data together, we found that OPLL cells express more ossification related genes than PLL cells.

To summarize and to visualize the findings, we constructed the GO tree graphs using the GO terms analyzed before. As shown in Fig. 3d, upregulation of genes mainly take place under the GO terms of extracellular matrix organization, embryonic skeletal system and ossification, while majority of other terms consist of downregulated genes. Together, we showed that OPLL altered genes were strongly correlated to ossification.

### Integrated microRNA/mRNA analysis implied OPLL specific microRNAs are important regulators in controlling OPLL gene alteration

**Figure 4:**
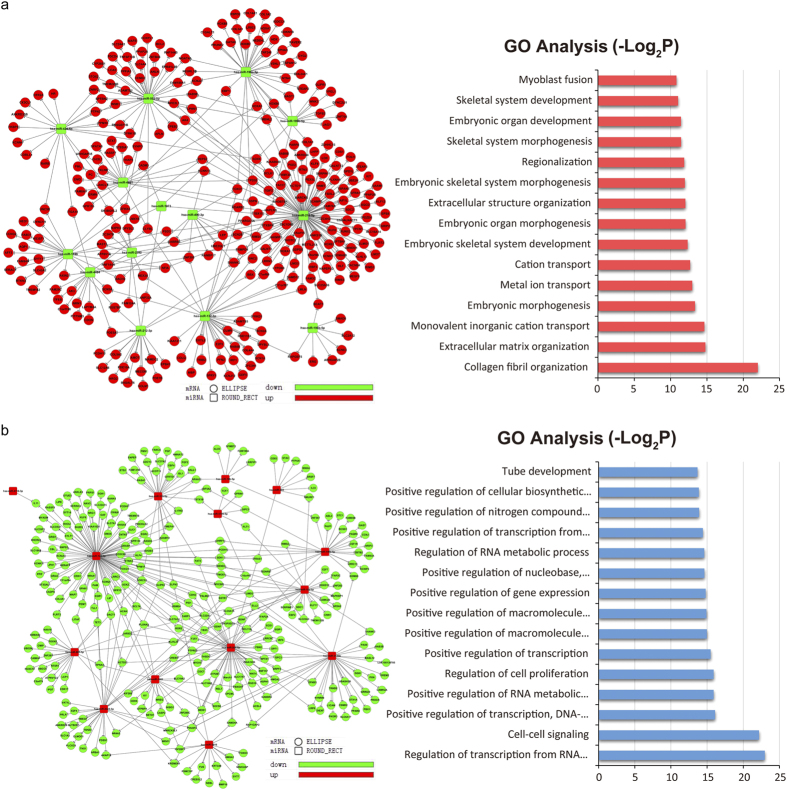
Integrated microRNA/mRNA network analysis. The differentially expressed miRNAs and mRNAs that identified were integrated by connecting miRNA with relevant mRNA using Targetscan miRNA targeting algorithm. These inversely correlated miRNA/mRNA pairs were sorted and the visualized networks were constructed using only the miRNAs with highest or lowest fold changes in OPLL compared to PLL. The correlation network showing 14 highly downregulated miRNAs and its possible target mRNAs were constructed (**a**). The GO analysis of these target genes were shown in the right panel. The correlation network showing 14 highly upregulated miRNAs and its possible target mRNAs were constructed (**b**). The GO analysis of these target genes were shown in the right panel.

To generate OPLL specific miRNA/mRNA interacting network, we used the OPLL specific miRNAs identified previously and predicted their target mRNAs using Targetscan (http://www.targetscan.org, Supplementary data 3). The predicted targets are overlapped with the transcriptome data, only genes with the expression pattern that negatively correlates with its targeting miRNAs are considered. MiRNAs with low abundance were also neglected (Normalized counts <100 in both groups). To scale down the whole network, we chose the top 15 regulated miRNAs as core miRNAs to generate the network. As shown in Fig. 4a, 14 top downregulated miRNAs and their matched mRNA targets were inversely correlated. In order to classify the related functions of these miRNA regulated genes a GO analysis was performed, results showed that these top upregulated targets were mainly associated with skeletal development and collagen fibril organization, which is similar to the results of whole transcriptome GO analysis. On the other hand, in the top 14 miRNAs and their matched targets were shown in Fig. 4b. And the GO analysis of these targeted downregulated genes also shown similar results to that of whole transcriptome GO analysis. Taken these together, we showed that the miRNA/mRNA network generated using these top regulated miRNAs can indeed represent most of the transcriptome changes, which implies that these miRNAs have set up the core transcription regulatory network in the OPLL.

**Figure 5:**
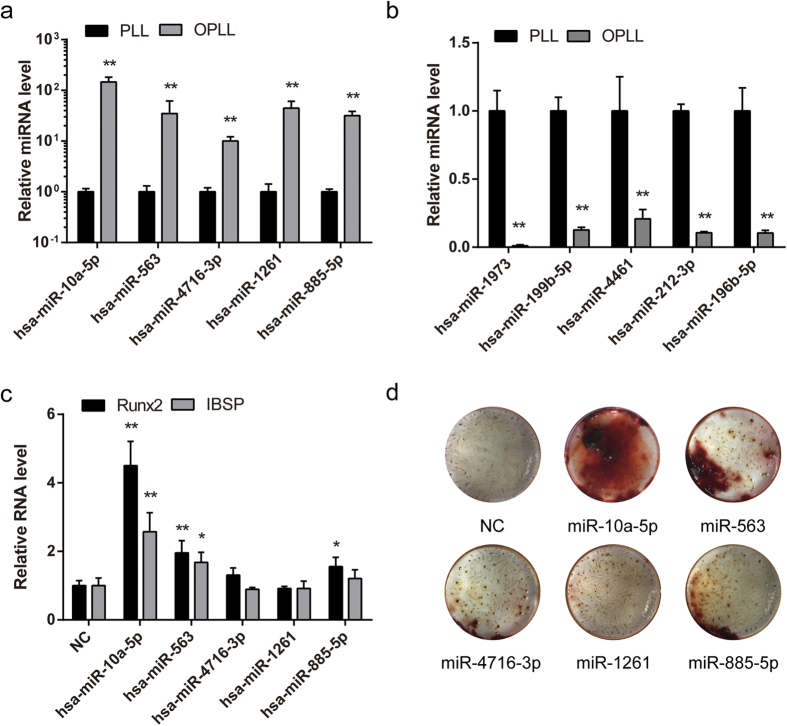
OPLL specific miRNAs regulate osteogenic differentiation of ligament cells. Five upregulated and five downregulated miRNAs were chosen for further verification. Their expressions in PLL and OPLL tissue samples were analyzed using real-time PCR analysis (**a,b**). Moreover, the function of five OPLL upregulated miRNAs were tested by overexpressing synthesized mimics and analyzed the expression changes of the osteogenic related factors Runx2 and Ibsp (**c**). Images of Alizarin Red S staining of the miRNA overexpressed PLL cells were shown to analyze the calcium deposition after osteo-induction for 2 weeks (**d**). Data were shown as mean ± S.D. and the P value are shown as *P < 0.05, **P < 0.01.

To unveil their importance in regulating longitudinal ligament cells transdifferentiating into osteogenic cells, we first verified the expression of 10 differentially expressed miRNAs using 4 PLL and 4 OPLL specimens. Real-time PCR identified the expression changes of these miRNAs between PLL and OPLL are similar to the sequencing results (Fig. 5a,b). To test their functions, we chose 5 most upregulated miRNAs and synthesized their mimics. After transfecting these mimics to PLL cells, we found that the expression of osteogenic related genes Runx2 and Ibsp were upregulated in some of the groups, especially in miR-10a-5p overexpressed group (Fig. 5c). Alizarin Red staining further showed that PLL cells can trans-differentiate into osteo-lineage, and that miR-10a-5p showed robust calcium deposition compared with a scramble RNA oligonucleotide overexpressed group as control (Fig. 5d). These results further implied that the OPLL differentially expressed miRNAs are indeed of biological significance in controlling OPLL onset and development.

## Discussion

The first report demonstrating OPLL can be traced back to 1960 s, which has a prevalence of 1.9–4.3% in the Japanese population^19^. Despite a long-standing predominance in Japan, this disease has also been recognized in other geographic regions and ethnicities, in the western regions the prevalence is only about 0.01–1.7%^20^. Recent study showed that the incidence rate of OPLL has reach more than 5% in some of the Asian regions outside Japan^21^. The disease also increases with age, with the average onset age of around 50 years old, and the male to female ratio at 2:1^22^. Despite advances in surgery and radiographic technologies, treating OPLL is still a task that has not been improved. Patients with serious symptoms that proceed to surgical treatment often resulted in unsatisfactory prognosis. Studies have been conducted to uncover the underlying factors in causing OPLL development, however, the factor seemed to be unspecific, indicating it’s an interplay of numerous genetic and environmental factors^23^. Beside non-genetic factors like mechanical stress, nutrition, glucose intolerance, and high body mass index, recent studies showed numerous genes are associated with the disease. Most of these genes are vital to the ossification process like NPPS, COL11A2, COL6A1, BMP2, BMP4, TGF-β1, TGF-β3^4^,^5^,^6^,^7^,^22^,^24^, and often aberrant expression of these genes can cause the pathological heterogeneous ossification^25^,^26^. This means a certain disorder in the upstream regulatory network of these critical factors may exists during the pathological process of OPLL.

There are many regulatory factors controlling certain genes’ expression, including transcriptional factors, epigenetic factors and post transcriptional factors^27^. Among which microRNA is the most well characterized post transcriptional factor that has been systematically researched in many biological fields. Owing to the high throughput technologies, the existence of miRNAs are greatly expanded and identified recently, with 2588 mature miRNAs identified in the latest version of miRBase. Beside the advancement of high throughput technologies, miRNA target prediction methods have also developed quickly. Although many algorithms were developed nowadays, the most widely used is Targetscan, which use algorithm that mainly depend on the seed sequence binding to the 3′ untranslated region of mRNA. With the identification of the crystal structure of miRNA binding protein Argonaute-2^28^,^29^, it is now widely accepted that this algorithm can predict functional miRNA interacting pairs. In the present study, we used Next Generation sequencing technology (NGS) to assess the expression change in both miRNA and mRNA, which generated more and accurate data than the traditional microarray assays. Altogether 1520 miRNAs were identified in both OPLL and PLL, while other microarray datasets we used in the study only generated about 700 miRNA data. Using integrative methods, we validated that 194 miRNAs that differentially expressed in OPLL are cell type specific, which generated OPLL specific miRNA signatures for the first time. These miRNA signatures may be involved in the onset and progression of OPLL and provide novel targets for OPLL diagnostic or therapeutic causes.

To investigate in depth of how these signature miRNAs really participates in the gene alteration of OPLL, we took advantage of the bioinformatics analysis. The Gene Ontology (GO) analysis is widely recognized as the leading tool for the organization and functional annotation of molecular attributes^30^. By using this tool, numerous differentially expressed genes in the transcriptome sequencing analysis were categorized into a few significant GO terms. Among the identified GO terms, skeletal system development, ossification, osteoblast differentiation have a relative low P value, which indicates that most upregulated genes in OPLL were related to such function or biological process. The result of which seemed to be reasonable for primary ligament cells derived from OPLL patients may already been influenced and prepared for ossification transformation in the genetic level. And this also gave direct evidence that the osteophytes in the OPLL patients may indeed come from untransformed posterior longitudinal ligament cells with proper stimulation. However, the genes that involved in these GO terms are also non-specific, one such example is Runx2 (About 3.7 fold increased in expression in OPLL than PLL), a well-defined master transcription factor that participates in almost every event involving ossification. Other ossification related genes like IBSP, collagen I and TGFB family were also upregulated in OPLL. Using high throughput datasets from existing databases, we are surprised to found that the miRNA signatures are specific to OPLL. And by constructing the miRNA/mRNA interacting network, we showed that these specific miRNA signatures can indeed drive the ossification related genes’ expression change during OPLL, which implies these specific signatures may be the key regulators that control and alter the transcriptome of ligament cells toward a more ossification ready state.

However, the clinical relevance of these specific signature miRNAs and their targeting pairs still need further validation. But still the study we presented here elucidated the altered mirNome and transcriptome, and identified predominant miRNA/mRNA interacting pairs that seemed to be of significance in the OPLL development. We are looking forward to further clarify their biological significance with further validation.

## Methods

### Sample collection and primary cell culture

The diagnosis of OPLL or PLL (spinal trauma patients who underwent cervical corpectomy) was confirmed using X-rays, computerized tomography, and magnetic resonance imaging preoperatively in the institution. OPLL or PLL specimens obtained during surgery and immediately put to primary cell culture. The ligaments were rinsed using PBS supplemented with 1% penicillin/streptomycin twice. After which the ligament tissue was carefully dissecting under microscope to avoid any contamination with osteogenic or other cells. The collected ligaments were minced and washed twice with PBS. Tissues were plated on a 100 mm culture dish and maintained in Dulbecco’s Modified Eagle Medium (DMEM, Life technology Gibco, USA) supplemented with 10% fetal bovine serum (FBS, Gibco, USA), 1% L-glutamine (Gibco, USA), and 1% penicillin / streptomycin (Gibco, USA). Tissues were incubated in a humidified atmosphere containing 95% air and 5% CO_2_ at 37 °C. The fibroblast-like cells that migrated from the tissues were harvested with 0.02% EDTA)/ 0.05% trypsin and plated in T25 culture flask for further analysis. The ethics committees of the Changzheng Affiliated Hospital of Second Military Medical University approved the study protocols, and each participant have written informed consent. The methods were carried out in accordance with the approved guidelines. We totally obtained 3 OPLL patient tissue samples during anterior cervical discectomy, and 3 PLL patient sample during anterior cervical corpectomy.

### Transcriptome and micronome profiling

For transcriptome sequencing, total RNA was extracted using the TRIzol solution (Invitrogen, Carlsbad, USA), according to the manufactures’ procotols. RNA sequencing libraries were constructed as described^31^ with some modifications. In brief, RNA concentration was measured by Nanodrop and the quality was measured by both gel electrophoresis and Agilent 2100. Samples that passed RNA quality control check will begin library preparation. We use Ion Total RNA-Seq Kit v2 (Life Technologies, USA) for library construction following manufacturer’s protocol. After purifying the poly-A containing mRNA molecules using poly-T oligo-attached magnetic beads, the mRNA is fragmented into small pieces using RNase III. The cleaved RNA fragments are concentrated again using the Nucleic Acid Binding Beads provided by the manufacturer. The purified RNA fragments are ligated and reverse transcribed into cDNA. The products are then purified and amplified with PCR (15-cycle) to create the final cDNA library. After purification, quantification and validation, validated cDNA libraries were sequenced on Ion Proton™ System (Ion Proton, Life Technologies, USA) following the manufacturer’s standard workflow. For small RNA sequencing, 10 μg total RNA of each sample was used for small RNA cDNA library preparation as previously described^32^ with some modifications. Strand-specific RNA libraries were prepared using the TruSeq Small RNA Sample Prep Kit (Illumina, San Diego, USA). Briefly, small RNA fragments ranging from 18–30 nt were isolated, purified and subsequently ligated were ligated to 3′ and 5′ adaptors sequentially, reverse transcribed to cDNA and then PCR amplified. The entire library was tested by gel electrophoresis, and bands corresponding to microRNA insertion were cut and eluted. After ethanol precipitation and washing, the purified small RNA libraries were quantified sequenced using the Illumina HiSeq™ 2000 analyzer (Illumina, San Diego, USA) according to the manufacturer’s instructions. All profiling works were done under the help of Shanghai NovelBio Bio-Pharm Technology Co., Ltd.

### Analysis of RNA sequencing data

For analysis of transcriptome sequencing data, we aligned approximately 100 bp long reads to the human genome (hg19) using Tophat2/Bowtie2^33^,^34^ allowing for five mismatches. We identified mapped data to gene structures derived from RefSeq using the summarize overlaps function with mode Intersect Strict (Genomic Ranges, Bioconductor). Using the initial raw counts data, we calculated reads per kilobase per million reads mapped (RPKM) values for the same gene set using Cufflinks. The differential analysis was carried out using edgeR^35^, applying TMM (trimmed Mean of M-values) library normalization and a 0.05 false discovery rate (FDR) to select expressed transcripts. For analysis of small RNA sequencing data, we first generated the clean reads and aligned with human genome (hg19) using Tophat2/Bowtie2^33^,^34^, followed by calculating the reads in different regions of the genome distribution. The clean reads were compared with the Rfam database (ftp://selab.janelia.org/pub/Rfam) to match the known lncRNA, miRNA, rRNA, snRNA, snRNA and tRNA sequences and were then compared with the human mature miRNAs database in miRBase (v21) to identify mature miRNAs and count their reads. The raw counts of miRNA reads were further normalized by transcripts per million (TPM) values ((miRNA total reads/total clean reads) ×10^6^). The differentially expressed miRNAs (DEmiRNAs) between samples were identified by the EdgeR program using parameters of P ≤ 0.01 and fold change ≥2 or ≤0.5.

### Gene Ontology and KEGG pathway analysis

Gene Ontology (GO) and KEGG pathway analyses were performed as previously described^36^. In brief, we calculated the p-value of each GO term using right-sided hypergeometric tests, and Benjamini-Hochberg adjustment was used for multiple test correction. An adjusted p-value that is lower than 0.05 indicated a statistically significant deviation from the expected distribution, and thus the corresponding GO terms and pathways were enriched in target genes. We analyzed all of the differentially expressed mRNAs using GO and KEGG pathway analyses, and we analyzed the mRNAs that were included in the miRNA regulator network using GO analysis.

### miRNA regulatory network construction

Targetscan was used to predict the binding of differentially expressed miRNAs to the putative targets. The predicted target genes were compared with the transcriptome profiling data, and only genes that are inversely correlated in expression with the targeting miRNA were included. After the correlation mapping, upregulated or downregulated miRNAs and their targeting genes were subjected to the network visualization analysis individually. The igraph package of the statistical language R was applied for functional profiling, and the network visualization and analysis tool Cytoscape22 was used to construct the network.

### Hierarchical clustering

Hierarchical clustering was performed as described^37^ using Cluster version 3.0 and Java TreeView version 1.1.6 to identify and visualize expression differences between the dataset. With the use of clustering algorithms, the samples and genes were grouped based on similarities in the expression profiles. For clustering of small RNA or mRNA sequencing data, we used normalized counts or RPKM for the analysis. Genes were filtered to exclude missing values. The average linkage and median centering were chosen, and unsupervised clustering were used. For clustering miRNA data of multiple cell types, we first downloaded the required data from GEO dataset under the Accession codes GSE19232, GSE34144 and GSE47025. The miRNA ID of each data were updated with miRbase v21 and thus can be integrated into a single dataset. Quintile normalization method was applied to scale each dataset to a uniform expression level. Unsupervised clustering using average linkage and median centering were sub sequentially performed.

### Real-time PCR and Alizarin Red staining

Total RNA were extracted by TRIzol solution (Invitrogen, Carlsbad, USA) and reverse transcribed using ReverTra Ace^®^ qPCR RT Kit (Toyobo, Osaka, Japan). Single strand cDNA were analyzed with SYBR Green master mix (Roche, USA) according to the instructions made by the manufacturer. Primers used in the real-time PCR were listed in the Supplementary dataset 5. Before staining Alizarin Red S (Sciencell, San Diego, USA), PLL cells were treated with osteo-induction medium consisting DMEM supplemented with 10% FBS, 25 mg/ml ascorbate-2 phosphate, 10^−8^ M dexamethasone, and 5 mM β-glycerophosphate (All from Gibco, USA) for 2 weeks. After induction, cells were fixed with 4% paraformaldehyde for 15 min, after two wash with PBS, cells were stained according to the manufacturer’s instruction.

## Additional Information

**Accession codes:** High throughput data including the transcriptome and micronome sequencing profiles of OPLL and PLL were submitted to the Gene Expression Omnibus database (GEO dataset). The accession number is GSE69787.

**How to cite this article**: Xu, C. *et al*. Integrated microRNA-mRNA analyses reveal OPLL specific microRNA regulatory network using high-throughput sequencing. *Sci. Rep.* **6**, 21580; doi: 10.1038/srep21580 (2016).

## Supplementary Material

Supplementary Information

Supplementary dataset 1

Supplementary dataset 2

Supplementary dataset 3

Supplementary dataset 4

Supplementary dataset 5

## Figures and Tables

**Table 1:** List of validated miRNAs that are differentially expressed in OPLL.

miRNA	Fold Change (OPLL/PLL)	P-Value	FDR	OPLL vs PLL*	MSC-osteo vs MSC*
Top 50 Upregulated miRNAs					
hsa-miR-10a-3p	630.71	1.56E-38	7.90E-36	Up-regulated	Down-regulated
hsa-miR-10a-5p	460.77	1.02E-88	1.55E-85	Up-regulated	Not significant
hsa-miR-371a-3p	247.28	3.75E-16	1.90E-14	Up-regulated	Not significant
hsa-miR-516a-5p	104.58	6.44E-13	2.45E-11	Up-regulated	Down-regulated
hsa-miR-563	93.94	1.00E-28	2.31E-26	Up-regulated	Not significant
hsa-miR-554	80.34	3.31E-05	4.09E-04	Up-regulated	Not significant
hsa-miR-4716-3p	69.72	6.32E-24	6.41E-22	Up-regulated	Not significant
hsa-miR-888-5p	61.26	3.37E-08	7.32E-07	Up-regulated	Not significant
hsa-miR-564	53.81	1.24E-03	8.86E-03	Up-regulated	Not significant
hsa-miR-582-3p	49.66	4.68E-07	8.28E-06	Up-regulated	Not significant
hsa-miR-551b-3p	37.62	1.06E-28	2.31E-26	Up-regulated	Not significant
hsa-miR-1261	34.51	2.51E-18	1.74E-16	Up-regulated	Not significant
hsa-miR-623	34.50	6.25E-05	7.15E-04	Up-regulated	Up-regulated
hsa-miR-885-5p	33.32	7.46E-28	1.42E-25	Up-regulated	Not significant
hsa-miR-620	33.31	1.30E-04	1.33E-03	Up-regulated	Not significant
hsa-miR-662	30.46	3.29E-07	6.18E-06	Up-regulated	Up-regulated
hsa-miR-498	29.51	5.37E-04	4.25E-03	Up-regulated	Down-regulated
hsa-miR-885-3p	21.23	1.96E-08	4.45E-07	Up-regulated	Not significant
hsa-miR-582-5p	20.19	2.78E-15	1.24E-13	Up-regulated	Down-regulated
hsa-miR-95-3p	19.27	9.14E-07	1.53E-05	Up-regulated	Down-regulated
hsa-miR-195-3p	17.32	1.82E-16	1.03E-14	Up-regulated	Not significant
hsa-miR-520d-3p	13.97	5.84E-06	8.87E-05	Up-regulated	Not significant
hsa-miR-657	13.27	6.66E-05	7.39E-04	Up-regulated	Down-regulated
hsa-miR-135a-5p	12.18	1.40E-04	1.39E-03	Up-regulated	Down-regulated
hsa-miR-526b-5p	11.33	6.40E-05	7.19E-04	Up-regulated	Down-regulated
hsa-miR-10b-5p	11.22	2.14E-22	2.03E-20	Up-regulated	Not significant
hsa-miR-204-5p	10.08	2.90E-19	2.10E-17	Up-regulated	Not significant
hsa-miR-622	9.92	1.97E-04	1.87E-03	Up-regulated	Not significant
hsa-miR-302a-5p	9.70	9.01E-05	9.60E-04	Up-regulated	Not significant
hsa-miR-569	8.58	3.17E-04	2.85E-03	Up-regulated	Not significant
hsa-miR-507	8.49	8.03E-04	6.07E-03	Up-regulated	Not significant
hsa-miR-210-3p	8.11	1.06E-17	6.73E-16	Up-regulated	Not significant
hsa-miR-516b-5p	7.67	1.00E-03	7.45E-03	Up-regulated	Not significant
hsa-miR-335-3p	6.72	2.38E-05	3.17E-04	Up-regulated	Down-regulated
hsa-miR-124-3p	6.37	9.11E-13	3.38E-11	Up-regulated	Down-regulated
hsa-miR-133a-3p	5.61	2.07E-04	1.95E-03	Up-regulated	Not significant
hsa-miR-147b	5.26	4.63E-07	8.28E-06	Up-regulated	Not significant
hsa-miR-548b-3p	4.81	1.25E-03	8.87E-03	Up-regulated	Down-regulated
hsa-miR-551a	4.61	6.44E-05	7.19E-04	Up-regulated	Down-regulated
hsa-miR-675-5p	4.53	9.09E-05	9.60E-04	Up-regulated	Not significant
hsa-miR-146a-5p	4.39	2.70E-10	7.61E-09	Up-regulated	Down-regulated
hsa-miR-483-5p	4.30	6.66E-07	1.15E-05	Up-regulated	Not significant
hsa-miR-335-5p	4.03	3.66E-09	9.44E-08	Up-regulated	Not significant
hsa-miR-628-3p	3.76	1.10E-03	8.05E-03	Up-regulated	Not significant
hsa-miR-181a-2-3p	3.70	2.07E-05	2.84E-04	Up-regulated	Not significant
hsa-miR-181a-5p	3.32	4.67E-05	5.54E-04	Up-regulated	Not significant
hsa-miR-10b-3p	3.30	1.35E-03	9.52E-03	Up-regulated	Not significant
hsa-miR-23b-3p	2.74	3.37E-05	4.10E-04	Up-regulated	Not significant
hsa-miR-137	2.71	4.66E-04	3.85E-03	Up-regulated	Not significant
hsa-miR-148a-3p	2.18	1.33E-03	9.41E-03	Up-regulated	Not significant
Top 50 Downregulated miRNAs					
hsa-miR-423-3p	0.50	8.57E-04	6.42E-03	Down-regulated	Not significant
hsa-miR-4521	0.49	3.34E-04	2.93E-03	Down-regulated	Not significant
hsa-miR-29a-5p	0.49	7.30E-04	5.57E-03	Down-regulated	Not significant
hsa-miR-199b-3p	0.48	4.91E-04	3.95E-03	Down-regulated	Not significant
hsa-miR-23a-5p	0.47	1.43E-03	9.94E-03	Down-regulated	Not significant
hsa-miR-31-3p	0.47	4.77E-04	3.89E-03	Down-regulated	Not significant
hsa-miR-324-3p	0.46	4.94E-04	3.95E-03	Down-regulated	Not significant
hsa-miR-16-2-3p	0.45	6.70E-04	5.17E-03	Down-regulated	Not significant
hsa-miR-149-5p	0.43	4.52E-04	3.76E-03	Down-regulated	Not significant
hsa-miR-330-3p	0.40	4.93E-04	3.95E-03	Down-regulated	Not significant
hsa-miR-31-5p	0.39	3.08E-05	3.84E-04	Down-regulated	Not significant
hsa-miR-887-3p	0.39	1.66E-04	1.62E-03	Down-regulated	Not significant
hsa-miR-337-5p	0.39	5.29E-05	6.14E-04	Down-regulated	Not significant
hsa-miR-708-5p	0.38	2.95E-05	3.77E-04	Down-regulated	Up-regulated
hsa-miR-625-3p	0.36	1.68E-05	2.39E-04	Down-regulated	Up-regulated
hsa-miR-34b-5p	0.35	2.65E-06	4.14E-05	Down-regulated	Not significant
hsa-miR-18a-5p	0.34	2.94E-06	4.57E-05	Down-regulated	Not significant
hsa-miR-130a-5p	0.33	7.66E-05	8.31E-04	Down-regulated	Down-regulated
hsa-miR-15b-3p	0.33	1.09E-05	1.57E-04	Down-regulated	Down-regulated
hsa-miR-34c-5p	0.32	4.01E-07	7.34E-06	Down-regulated	Not significant
hsa-miR-486-3p	0.31	4.78E-04	3.89E-03	Down-regulated	Not significant
hsa-miR-625-5p	0.31	1.52E-06	2.46E-05	Down-regulated	Not significant
hsa-miR-450b-5p	0.30	2.26E-04	2.12E-03	Down-regulated	Not significant
hsa-miR-222-5p	0.28	2.40E-08	5.36E-07	Down-regulated	Not significant
hsa-miR-542-3p	0.27	2.67E-07	5.20E-06	Down-regulated	Not significant
hsa-miR-146b-5p	0.23	1.80E-10	5.15E-09	Down-regulated	Not significant
hsa-miR-129-5p	0.22	1.40E-04	1.39E-03	Down-regulated	Not significant
hsa-miR-424-5p	0.20	4.68E-12	1.51E-10	Down-regulated	Not significant
hsa-miR-218-5p	0.19	1.86E-12	6.43E-11	Down-regulated	Not significant
hsa-miR-1246	0.19	1.99E-05	2.79E-04	Down-regulated	Not significant
hsa-miR-29b-1-5p	0.19	2.08E-13	8.09E-12	Down-regulated	Not significant
hsa-miR-132-3p	0.16	5.99E-14	2.40E-12	Down-regulated	Not significant
hsa-miR-196a-5p	0.16	3.90E-14	1.60E-12	Down-regulated	Not significant
hsa-miR-132-5p	0.15	7.67E-12	2.38E-10	Down-regulated	Not significant
hsa-miR-218-1-3p	0.15	1.20E-12	4.24E-11	Down-regulated	Not significant
hsa-miR-503-5p	0.15	2.56E-16	1.34E-14	Down-regulated	Not significant
hsa-miR-27a-5p	0.15	1.33E-14	5.62E-13	Down-regulated	Not significant
hsa-miR-490-3p	0.15	1.19E-08	2.78E-07	Down-regulated	Not significant
hsa-miR-424-3p	0.12	1.96E-15	9.04E-14	Down-regulated	Down-regulated
hsa-miR-196a-3p	0.12	3.26E-07	6.18E-06	Down-regulated	Not significant
hsa-miR-92a-2-5p	0.11	5.06E-05	5.92E-04	Down-regulated	Not significant
hsa-miR-2392	0.09	6.37E-05	7.19E-04	Down-regulated	Not significant
hsa-miR-490-5p	0.08	6.03E-04	4.73E-03	Down-regulated	Not significant
hsa-miR-196b-5p	0.07	1.80E-26	2.49E-24	Down-regulated	Not significant
hsa-miR-212-3p	0.07	1.20E-19	9.12E-18	Down-regulated	Not significant
hsa-miR-129-2-3p	0.05	5.26E-22	4.70E-20	Down-regulated	Not significant
hsa-miR-4461	0.05	1.14E-04	1.19E-03	Down-regulated	Not significant
hsa-miR-199b-5p	0.04	9.77E-22	8.25E-20	Down-regulated	Not significant
hsa-miR-378b	0.02	8.08E-08	1.64E-06	Down-regulated	Not significant
hsa-miR-1973	0.01	3.45E-04	2.98E-03	Down-regulated	Not significant

^*^The fold changes of these differentially expressed miRNAs were calculated, and either ≥2 or ≤0.5 fold were considered as significant and labeled as up or down regulated according to the samples.
