# Supplementary material for: Integrated microRNA-mRNA analyses reveal OPLL specific microRNA regulatory network using high-throughput sequencing
Source: Sci Rep. 2016 Feb 12;6:21580. doi: 10.1038/srep21580 (PMC4751494; doi:10.1038/srep21580)
Supplement: Supplementary Information [file srep21580-s1.doc]

Supplementary information for

**Integrated microRNA-mRNA analyses reveal OPLL specific microRNA regulatory network using high-throughput sequencing**

**Authors:** Chen Xu^1,+^, Yu Chen^1,+^, Hao Zhang^1,+^, Yuanyuan Chen^1^, Xiaolong Shen^1^, Changgui Shi^1^, Wen Yuan^1,*^, and Yang Liu^1,*^

**Affiliations:** ^1^ Department of Orthopedics, Changzheng Hospital Affiliated to Second Military Medical University, 415th Feng Yang Road, Shanghai 200003, PR China.

^*^ Corresponding authors

^+^ These authors contributed equally to this work

**Contact Information of Corresponding Authors:**

Prof. Yang Liu, Tel and Fax: +86-021-81870958, E-mail: [liuyspine@126.com](mailto:liuyspine@126.com)

Prof. Wen Yuan, Tel and Fax: +86-021-81870958, E-mail: wenyuan@126.com

**Supplementary Figures**

**
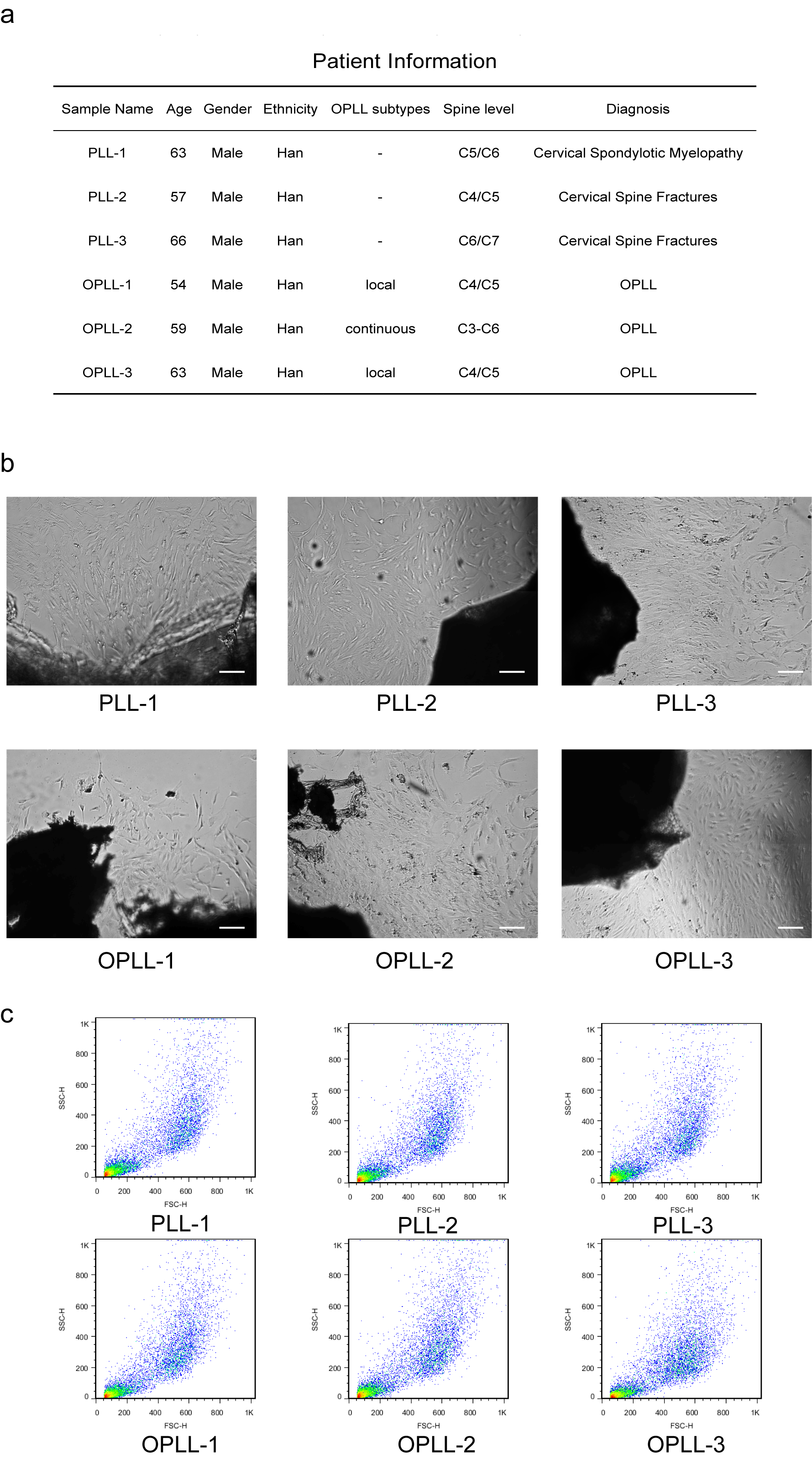
**

**Supplementary Figure 1.** Patient information of the analyzed samples (a) and the representative microscopy images of primary culture ligament cells of all samples (b). A flow cytometry analysis showing that the ligament cells are of same characteristics (c). The scale bar indicates 50μm.

**Supplementary Datasets**

**Supplementary Dataset 1.** The expression ranking of OPLL differentially expressed miRNAs in multiple cell types.

**Supplementary Dataset 2.** Gene Ontology analysis of differentially expressed mRNAs in OPLL.

**Supplementary Dataset 3.** The miRNA/mRNA pairs identified by integrating miRNA and mRNA profiling data using Targetscan.

**Supplementary Dataset 4.** List of target mRNAs of differentially expressed miRNAs in OPLL using Targetscan

**Supplementary Dataset 5.** List of primers used in the study
